# Mangiferin Protects Human Dermal Fibroblasts Against UVB-Induced Photoaging by Regulating RAGE/NF-κB/p38 MAPK Signaling, Cellular Senescence, and ECM Homeostasis

**DOI:** 10.3390/cimb48080757

**Published:** 2026-07-25

**Authors:** İnci Kurt-Celep

**Affiliations:** Department of Biotechnology, Faculty of Pharmacy, Istanbul Okan University, Tuzla, Istanbul 34959, Turkey; inci.celep@okan.edu.tr

**Keywords:** mangiferin, UVB-induced photoaging, DNA damage, cellular senescence, RAGE/NF-κB signaling, extracellular matrix degradation

## Abstract

Ultraviolet B (UVB) radiation is a major environmental factor contributing to skin photoaging through excessive reactive oxygen species (ROS) generation, activation of stress-responsive signaling pathways, DNA damage, cellular senescence, and extracellular matrix (ECM) degradation. Mangiferin, a naturally occurring xanthone glucoside with potent antioxidant and anti-inflammatory properties, has attracted considerable interest as a potential photoprotective agent. The present study investigated the protective effects of mangiferin against UVB-induced photoaging in human dermal fibroblasts (HDFs). The effects of mangiferin on oxidative stress, RAGE/NF-κB/MAPK signaling, DNA damage, cellular senescence, and ECM degradation were evaluated. Mangiferin significantly suppressed UVB-induced ROS accumulation and attenuated activation of the RAGE/NF-κB/MAPK signaling cascade. Furthermore, mangiferin reduced γ-H2AX expression, indicating protection against UVB-mediated DNA damage, while decreasing p16, p21, and p53 expression and restoring LMNB1 levels. Mangiferin also inhibited MMP-2 and MMP-9 activities as well as collagenase, elastase, and hyaluronidase activities, suggesting preservation of ECM homeostasis. These findings demonstrate that mangiferin protects dermal fibroblasts against UVB-induced photoaging through suppression of oxidative stress, inhibition of RAGE/NF-κB/MAPK signaling, attenuation of DNA damage and cellular senescence, and preservation of ECM integrity, supporting its potential application in photoprotective and anti-photoaging dermocosmetic formulations.

## 1. Introduction

Skin aging is a complex biological process resulting from the interaction of intrinsic (chronological) and extrinsic factors [1]. One of the most important causes of extrinsic aging is exposure to ultraviolet (UV) radiation, and this process is generally described as photoaging [2]. Ultraviolet B (UVB) radiation, a major component of sunlight, contributes to the acceleration of skin aging by causing various structural and functional changes in the epidermis and dermis. UVB exposure results in a decrease in collagen fibers, irregularities in elastic fibers, disruption of extracellular matrix (ECM) integrity, and changes in fibroblast function [3]. Since dermal fibroblasts are the primary cells responsible for the synthesis of collagen and other matrix components, molecular changes occurring in these cells are central to the photoaging process [4]. One of the primary mechanisms of UVB-induced photodamage is the overproduction of reactive oxygen species (ROS) [5]. Increased intracellular ROS levels disrupt redox balance, causing oxidative damage to lipids, proteins, and nucleic acids. Oxidative stress not only directly damages cellular components but also triggers the activation of numerous signaling pathways associated with aging [6]. The resulting chronic stress response contributes to impaired fibroblast function and loss of ECM homeostasis. Therefore, controlling UVB-induced oxidative stress is considered a crucial goal in preventing photoaging [7]. Recent studies investigating the molecular mechanisms underlying UVB-induced damage have indicated that advanced glycation end product receptor (RAGE)-mediated signal transduction networks play a significant role [8]. RAGE activation leads to the stimulation of various cellular signaling pathways, primarily nuclear factor kappa B (NF-κB) and mitogen-activated protein kinases (MAPKs). In particular, activation of the p38 MAPK and NF-κB pathways is associated with a strengthening of the inflammatory response, an increase in matrix metalloproteinases (MMPs), and disruption of ECM integrity. Therefore, the RAGE/NF-κB/p38 MAPK axis is considered one of the important molecular targets playing a role in the photoaging process [8]. Another significant consequence of UVB exposure is DNA damage. UVB-induced oxidative stress and genotoxic effects disrupt cellular DNA integrity, leading to the activation of DNA damage response mechanisms [9]. γ-H2AX, the phosphorylated form of the histone H2AX protein, is considered one of the most sensitive markers of DNA damage response and is widely used, particularly in the assessment of double-strand break signaling. Permanent DNA damage contributes to cell cycle arrest and the development of cellular senescence over time. Therefore, the assessment of DNA damage and associated cellular responses is of great importance for understanding the mechanisms of photoaging [10,11]. Cellular senescence is a biological state in which proliferative capacity is irreversibly lost, leading to age-related phenotypic changes. DNA damage response and oxidative stress mechanisms activated by UVB exposure contribute to the orientation of fibroblasts toward the senescence axis by activating cell cycle regulators such as p53, p21, and p16 [12,13]. Furthermore, changes in lamin B1 levels, a key component of the nuclear lamina, are also reported to be closely associated with cellular senescence [14]. Increased fibroblast senescence not only leads to decreased cellular proliferation and biosynthetic activity but also contributes to the disruption of mechanisms responsible for collagen production, ECM organization, and tissue remodeling [4,15]. This situation is considered one of the key determinants of the photoaging process by accelerating the loss of dermal matrix integrity. One of the significant consequences of the photoaging process is the loss of extracellular matrix homeostasis. Increased MMP activity following UVB exposure accelerates the degradation of collagen and other matrix components. In particular, the activation of gelatinases such as MMP-2 and MMP-9 plays a significant role in the disruption of dermal matrix integrity [4,15,16]. In addition, changes in the activity of enzymes such as collagenase, elastase, and hyaluronidase also contribute to the negative impact on skin structure and function. Therefore, maintaining ECM homeostasis is considered one of the fundamental goals of new approaches to combat photoaging [17].

Mangiferin is most extensively obtained from *Mangifera indica*, particularly its leaves and bark, although mango peel and seed kernels and the roots of *Salacia* species also represent documented natural sources. Conventional recovery commonly involves aqueous, alcoholic, or hydroalcoholic extraction using maceration, reflux, or Soxhlet procedures. Ultrasound- and microwave-assisted extraction methods have also been developed to improve extraction efficiency while reducing extraction time and solvent consumption. Following extraction, mangiferin may be enriched or purified using liquid–liquid fractionation, column chromatography, or preparative high-performance liquid chromatography [18,19]. In various experimental models, mangiferin has been reported to have positive effects on reducing oxidative stress, suppressing inflammatory signaling pathways, and limiting cellular damage [18,19]. However, studies that comprehensively evaluate the effects of mangiferin in a UVB-induced human dermal fibroblast photoaging model through mechanisms related to the RAGE/NF-κB/p38 MAPK signaling axis, DNA damage response, cellular senescence, and extracellular matrix homeostasis are limited [18]. This study aimed to investigate the protective effects of mangiferin in a UVB-induced human dermal fibroblast photoaging model. Specifically, the study aimed to evaluate its effects on cell viability, intracellular ROS levels, the RAGE/NF-κB/p38 MAPK signaling axis, γ-H2AX-mediated DNA damage response, cellular senescence markers, and extracellular matrix homeostasis.

## 2. Materials and Methods

### 2.1. Chemicals and Reagents

The mangiferin, dimethyl sulfoxide (DMSO), Dulbecco’s Modified Eagle Medium (DMEM), fetal bovine serum (FBS), penicillin–streptomycin solution, phosphate-buffered saline (PBS), and trypsin-EDTA solution used in this study were obtained from Sigma-Aldrich (St. Louis, MO, USA). All other chemicals used in the study were of analytical purity and obtained from commercial sources. The electrophoresis, transfer, and imaging systems used in Western blot analyses were obtained from Bio-Rad Laboratories (Hercules, CA, USA). Chemiluminescence signals of protein bands were captured using the ChemiDoc™ Imaging System (Bio-Rad Laboratories, Hercules, CA, USA). Densitometric analyses of membrane images were performed using the Gel Analysis tool of ImageJ software, version 1.54p (National Institutes of Health, Bethesda, MD, USA).

### 2.2. Cell Culture, Experimental Design and UVB Application

Human dermal fibroblast (HDF) cells were cultured in DMEM containing 10% fetal bovine serum and 1% penicillin–streptomycin at 37 °C, 5% CO_2_ and 95% humidity. When the HDF cells reached approximately 80–90% confluence, they were passaged using trypsin-EDTA, and commercially immortalized cells (PCS-201-012) were purchased from the American Type Culture Collection (ATCC, Manassas, VA, USA). In this study, the cells were divided into the following groups: control group (untreated HDF cells), UVB group, and Mangiferin + UVB group.

Cells to be treated with mangiferin were incubated with mangiferin at the highest non-cytotoxic working concentration obtained from WST-1 results for 48 h before UVB exposure. The cells to be treated with UVB application (HDF + UVB) were previously incubated with mangiferin at a concentration obtained from the WST-1 results. To generate the HDF + UVB cell model, a previously established UVB irradiation protocol was used [20,21]. In this context, HDF cells were seeded on a 6-well plate at a density of 1 × 10^6^, and after attachment, the cells were exposed to UVB light with a UVB crosslinker at a power of 0.5 J/cm^2^ for three minutes [20,21]. Also, the cell-based and biochemical experimental protocols were performed according to previously published methods [20,21,22,23], with assay-specific modifications and experimental conditions described in the corresponding subsections.

### 2.3. Cell Viability and Proliferation Analysis with WST-1

The effects of mangiferin on the viability and proliferation of HDF cells were evaluated using the WST-1 method. Cells were seeded into 96-well plates and treated according to the experimental groups. At the end of the incubation period, WST-1 solution was added to the wells, and absorbance values were measured at 450 nm using a microplate reader. Results were expressed as percentage viability compared to the untreated HDF group [20,21].

### 2.4. Determination of Intracellular ROS Levels

The effects of mangiferin on intracellular reactive oxygen species (ROS) in UVB-induced HDF cells were evaluated using the DCFDA method. For this purpose, HDF cells were divided into control, UVB, and UVB groups treated with mangiferin at the highest non-cytotoxic working concentration determined by WST-1 analysis. The cells were incubated for 48 h after the experimental applications and then treated with 2′,7′-dichlorodihydrofluorescein diacetate (DCFDA; Sigma-Aldrich, St. Louis, MO, USA) in a dark environment. The resulting fluorescence signal was measured at 485 nm excitation and 535 nm emission wavelengths in a microplate reader, and the results were expressed as relative fluorescence units (RFU) and evaluated in relation to the control group [22].

### 2.5. Western Blot Analysis

RAGE, NF-κB p65, phosphorylated p38 MAPK (p-p38), and γ-H2AX protein levels were evaluated using Western blot. Total protein extraction from cells was performed using lysis buffer containing protease and phosphatase inhibitors. Protein amounts were determined using the Bradford method. Equal amounts of protein were separated by SDS-PAGE and then transferred to PVDF membranes. After blocking, the membranes were incubated overnight at 4 °C with RAGE, NF-κB p65, p-p38 MAPK, γ-H2AX, and β-actin primary antibodies. All primary antibodies used were obtained from Cell Signaling Technology (Danvers, MA, USA). Subsequently, incubation was performed with HRP-conjugated secondary antibodies, and protein bands were captured using the ChemiDoc™ Imaging System (Bio-Rad Laboratories, Hercules, CA, USA). Target protein levels were normalized to β-actin loading control and analyzed using ImageJ software [22].

### 2.6. RNA Isolation and RT-qPCR Analysis for Senescence-Related Gene Detection

Total RNA isolation was performed using a commercially available PeqGold Trizol RNA isolation kit, following the manufacturer’s protocol. After spectrophotometric determination of RNA purity and concentration, 1000 ng of complementary DNA (cDNA) was synthesized from the obtained RNA samples. Expression levels of CDKN2A (p16INK4a), CDKN1A (p21), TP53, and LMNB1 genes were determined using real-time quantitative polymerase chain reaction (RT-qPCR). All primers were purchased from QIAGEN. The 18S rRNA was used as the reference (housekeeping) gene in gene expression analyses. Relative gene expression levels were calculated by ratioing them to 18S rRNA, with Untreated-HDFs considered as 1-fold in all gene analyses [22,23].

### 2.7. Gelatin Zymography Assay

MMP-2 and MMP-9 enzyme activities were evaluated using gelatin zymography. Conditioned medium samples obtained from the experimental groups were subjected to electrophoresis on 12% polyacrylamide gels containing 1% gelatin but without sodium dodecyl sulfate (SDS). After renaturation and incubation, the gels were stained with Coomassie Brilliant Blue. Regions showing gelatinolytic activity were evaluated as transparent bands, and densitometric analysis was performed using Image J software [20,22,23].

### 2.8. Collagenase, Elastase, and Hyaluronidase Inhibition Analyses

The inhibitory effects of mangiferin on collagenase, elastase, and hyaluronidase enzymes were evaluated using spectrophotometric methods. For collagenase and elastase analyses, -(3-[2-Furyl]acryloyl)-Leu-Gly-Pro-Ala (FALGPA) and N-Succinyl-Ala-Ala-Ala-p-nitroanilide (AAAPVN) substrates were used, respectively; absorbance changes were measured at 340 nm and 410 nm, respectively. Epigallocatechin gallate (EGCG) was used as a positive control in both analyses. Hyaluronidase inhibition was evaluated using bovine testis-derived enzyme (EC 3.2.1.35; Sigma-Aldrich, St. Louis, MO, USA); the resulting turbidity was measured at 600 nm, and the results were compared with tannic acid used as a positive control. Enzyme inhibition percentages were calculated based on the percentage inhibition compared to each experimental group’s own standard [22].

### 2.9. Statistical Analysis

The results were expressed as mean ± standard deviation (SD). Statistical analyses were carried out using GraphPad Prism software, version 8.4.2 (GraphPad Software, San Diego, CA, USA). Cell viability data obtained from the WST-1 assay were analyzed using one-way analysis of variance (One-Way ANOVA) followed by Dunnett’s multiple comparisons test. Data from intracellular ROS measurements, Western blot densitometric analyses, RT-qPCR experiments, gelatin zymography assays, and ECM-related enzyme inhibition assays were analyzed using one-way ANOVA followed by Tukey’s multiple comparisons test. A *p*-value ≤ 0.05 was considered statistically significant [22].

## 3. Results

### 3.1. Effects of Mangiferin on HDF Cell Viability

**Figure 1:**
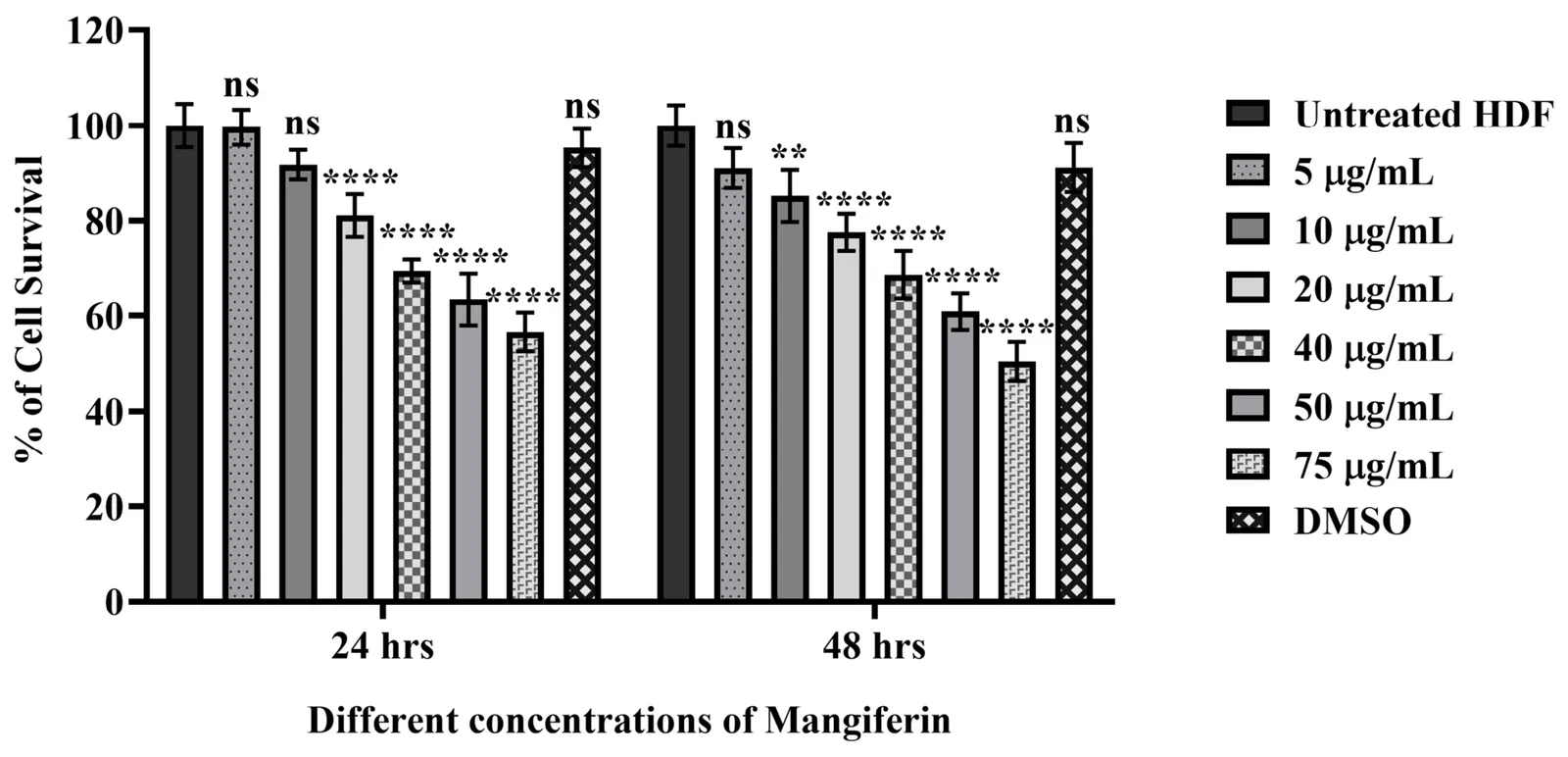
Effects of mangiferin on the viability of human dermal fibroblasts (HDFs) following 24 and 48 h treatment, as determined by the WST-1 assay. Cell viability was expressed as a percentage relative to untreated HDF cells. Results were presented as mean ± SD. Statistical analysis was performed using one-way ANOVA followed by Dunnett’s multiple comparisons test. ns, not significant; ** *p* ≤ 0.01; **** *p* ≤ 0.0001 versus untreated HDF cells.

The concentration-dependent effects of mangiferin on the viability of HDF cells were determined using the WST-1 method, and the results at 24 and 48 h were presented in Figure 1. Cell viability decreased from 99.64% ± 3.70 (ns) to 56.60% ± 4.01 (**** meaning *p* ≤ 0.0001) at 24 h and from 91.04% ± 4.15 (ns) to 50.45% ± 4.05 (**** meaning *p* ≤ 0.0001) at 48 h in the concentration range of 5–75 µg/mL. In the vehicle control group containing 0.1% DMSO, cell viability was determined as 95.27% ± 4.01 and 91.18% ± 5.13 at 24 and 48 h, respectively (ns). Based on the WST-1 results obtained at 24 and 48 h, 50 µg/mL was selected as the highest non-cytotoxic working concentration because relative cellular viability remained above 60% at both evaluated time points. In contrast, treatment with 75 µg/mL mangiferin reduced relative viability to 56.60% ± 4.01 at 24 h and 50.45% ± 4.05 at 48 h. The marked reduction observed at 48 h was particularly important because all protein lysates, RNA/cDNA samples, and conditioned media used in the subsequent molecular analyses were collected at the end of the 48 h experimental period. At this concentration and time point, only approximately half of the metabolic viability of untreated cells was preserved. Therefore, 75 µg/mL was excluded to avoid potential cytotoxic or cytostatic interference that could confound the interpretation of protein levels, gene expression, and MMP activity, and 50 µg/mL was used in all subsequent cellular experiments [22,24].

### 3.2. Photoprotective Effect of Mangiferin Against UVB-Induced Intracellular Oxidative Stress

**Figure 2:**
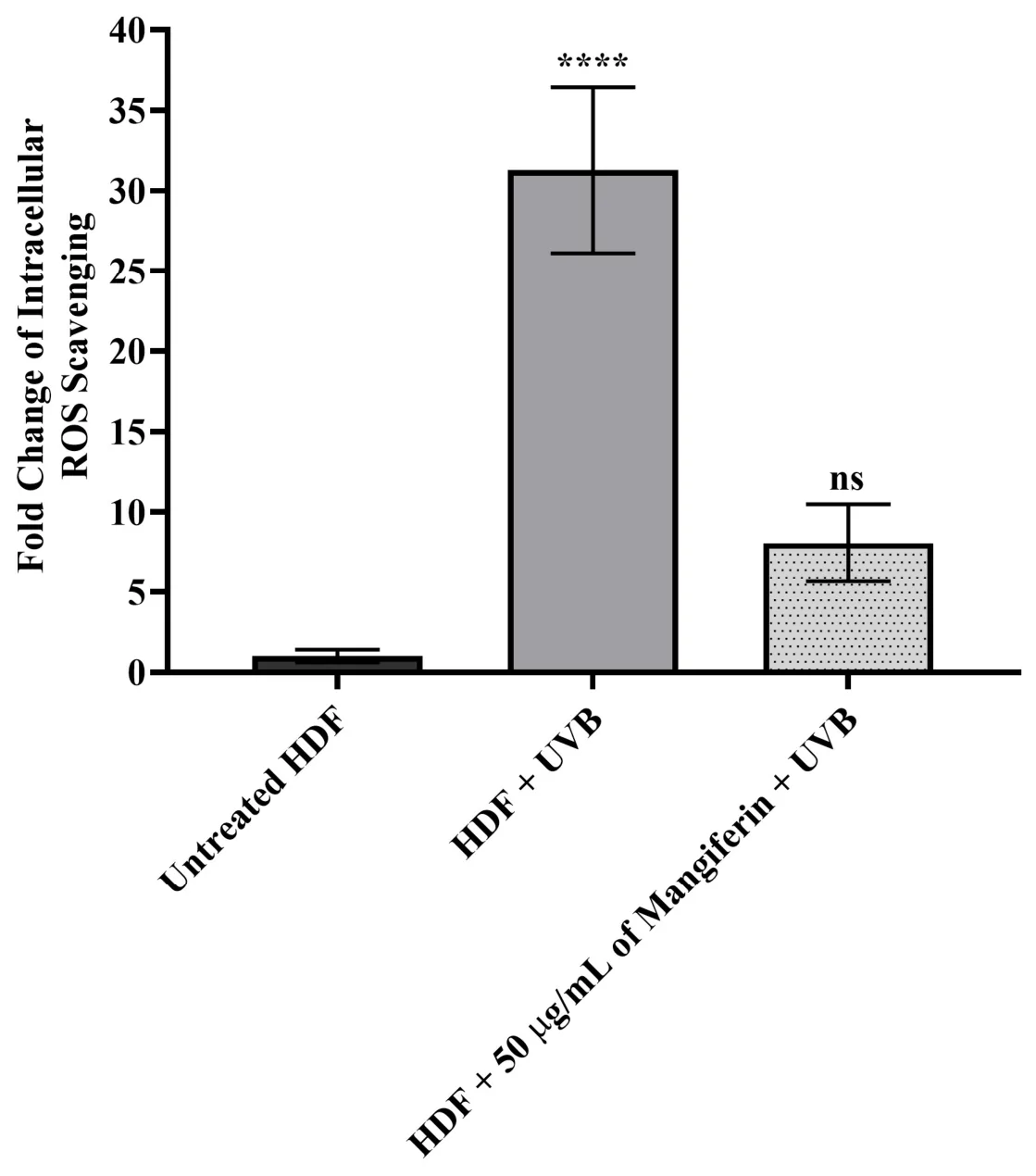
Effects of mangiferin on intracellular reactive oxygen species (ROS) levels in UVB-exposed human dermal fibroblasts (HDFs), as determined by the DCFDA assay. Intracellular ROS levels are expressed as fold change relative to untreated HDF cells. Data was presented as mean ± SD. Statistical analysis was performed using one-way ANOVA followed by Tukey’s multiple comparisons test. ns, not significant versus untreated HDF cells; **** *p* ≤ 0.0001 versus untreated HDF cells.

The protective effect of mangiferin against UVB-induced oxidative stress was evaluated by measuring intracellular ROS levels in HDF cells using the DCFDA method (Figure 2). UVB exposure caused a significant increase in intracellular ROS levels compared to the control group, with a relative fluorescence value rising from 1.00 ± 0.40 to 31.28 ± 5.16 (*p* ≤ 0.0001). In contrast, in cells pre-treated with 50 µg/mL mangiferin before UVB application, the ROS level was determined as 8.07 ± 2.41, which was significantly lower than the UVB group (*p* ≤ 0.0001 indicated ****). These findings indicate that mangiferin contributes to the maintenance of cellular redox balance by suppressing excessive ROS formation triggered by UVB exposure.

### 3.3. Effects of Mangiferin on UVB-Induced Signal Transmission and DNA Damage

**Figure 3:**
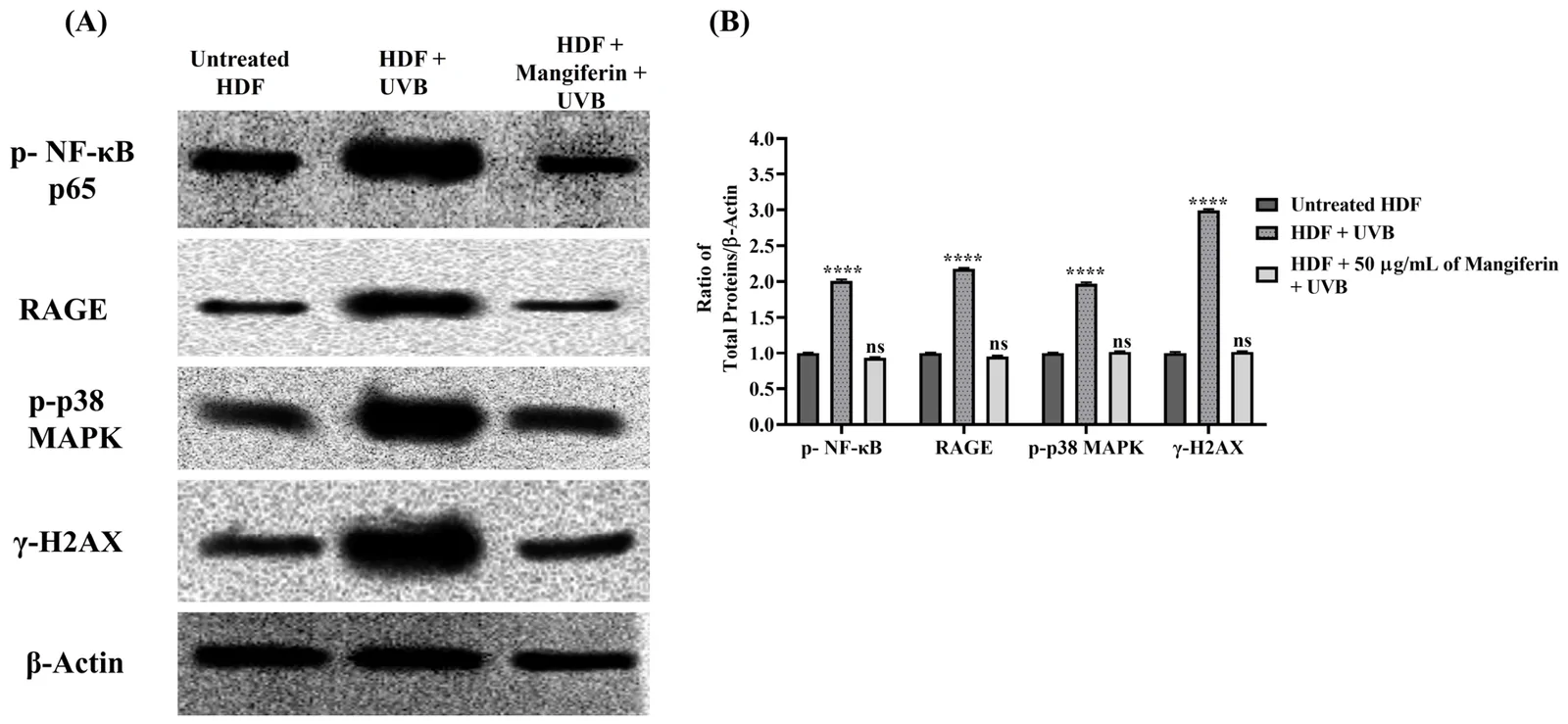
Effects of mangiferin on UVB-induced activation of RAGE/NF-κB/p38 MAPK signaling and DNA damage in human dermal fibroblasts (HDFs). (**A**) Representative Western blot images showing the expression levels of phosphorylated NF-κB p65 (p-NF-κB p65), RAGE, phosphorylated p38 MAPK (p-p38 MAPK), and γ-H2AX in untreated HDFs, UVB-exposed HDFs, and mangiferin-pretreated UVB-exposed HDFs. β-Actin was used as the loading control. (**B**) Densitometric analysis of protein expression levels normalized to β-actin and expressed as fold change relative to untreated HDF cells. Data were presented as mean ± SD of three independent experiments (n = 3). Statistical analysis was performed using one-way ANOVA followed by Tukey’s multiple comparisons test. ns, not significant; **** *p* ≤ 0.0001 versus untreated HDF cells.

Western blot analyses revealed that UVB treatment activated the RAGE/NF-κB/p38 MAPK signaling pathway in human dermal fibroblasts and increased the level of γ-H2AX protein, a key indicator of DNA damage (Figure 3). Compared to untreated HDF cells, UVB exposure resulted in p-NF-κB, RAGE, p-p38 MAPK, and γ-H2AX protein levels increasing 2.01-fold, 2.18-fold, 1.97-fold, and 2.99-fold, respectively (*p* ≤ 0.0001 displayed ****). The most significant increase was observed in γ-H2AX protein. On the other hand, in cells exposed to UVB following pre-treatment of mangiferin, the levels of these proteins were significantly suppressed. In the group treated with mangiferin, p-NF-κB, RAGE, p-p38 MAPK, and γ-H2AX protein levels were found to be 0.93-fold, 0.95-fold, 1.02-fold, and 1.02-fold, respectively (ns). Compared to the UVB group, mangiferin treatment caused a decrease of approximately 53.7%, 56.4%, 48.2%, and 65.9% in p-NF-κB, RAGE, p-p38 MAPK, and γ-H2AX protein levels, respectively, as shown in the results presented in Figure 3B (**** meaning *p* ≤ 0.0001). These findings demonstrate that mangiferin suppresses UVB-induced inflammatory signal activation and exhibits a protective effect against DNA damage.

### 3.4. Effects of Mangiferin on Gene Expressions Associated with UVB-Induced Cellular Senescence

**Figure 4:**
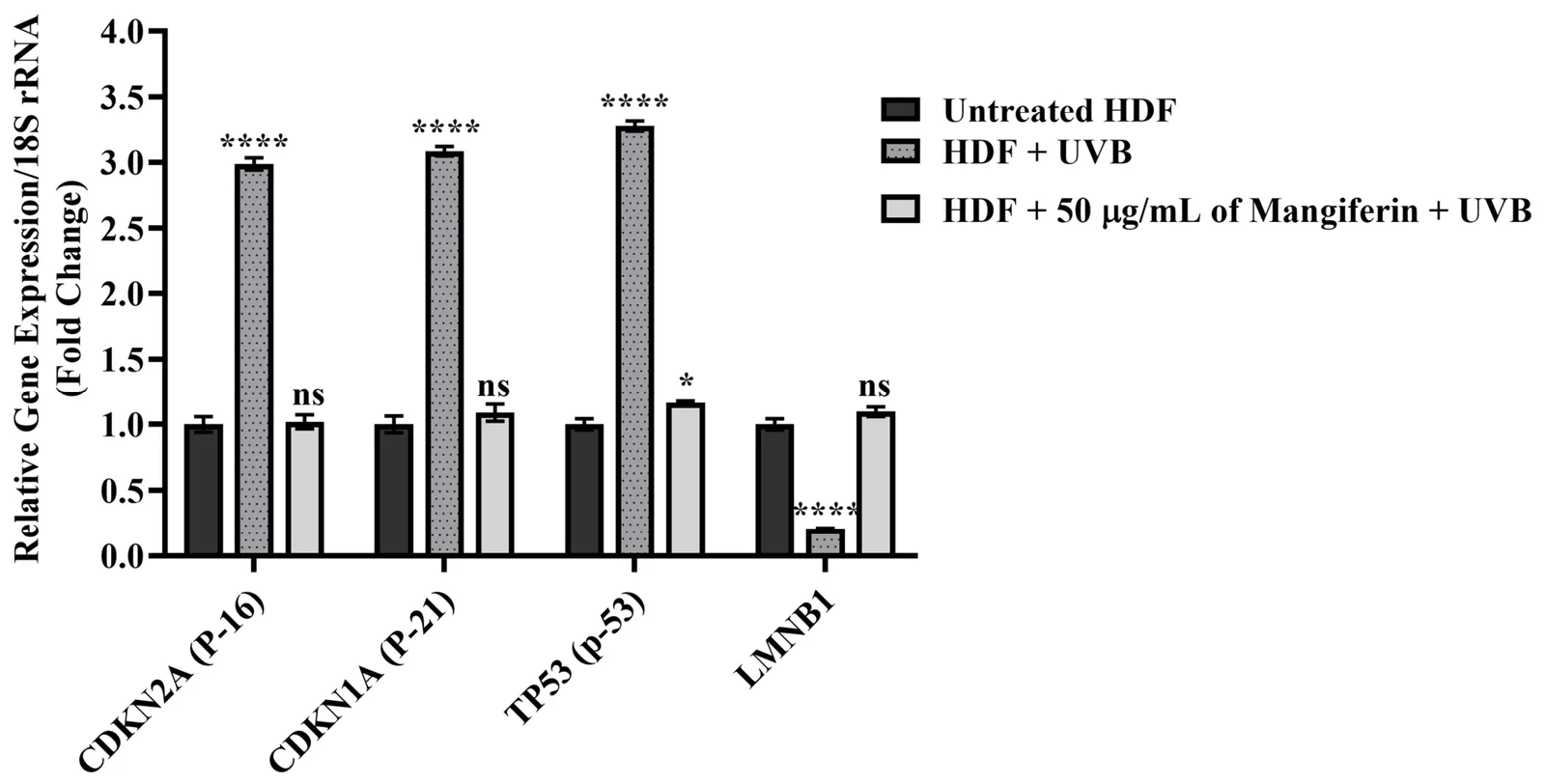
Effects of mangiferin on UVB-induced senescence-associated gene expression in human dermal fibroblasts (HDFs). Relative mRNA expression levels of CDKN2A (P-16), CDKN1A (P-21), TP53 (p-53), and LMNB1 were determined by RT-qPCR and normalized to 18S rRNA. Data were expressed as mean ± SD. Statistical analysis was performed using one-way ANOVA followed by Tukey’s multiple comparisons test. ns, not significant; * *p* ≤ 0.05; **** *p* ≤ 0.0001 versus untreated HDF cells.

The effects of UVB exposure and mangiferin pre-treatment on the expression levels of senescence-related genes were evaluated using the RT-qPCR method, and the results are presented in Figure 4. UVB treatment caused significant increases in the expression of CDKN2A, CDKN1A, and TP53 genes compared to the control group, with expression levels determined as 2.99-fold, 3.08-fold, and 3.27-fold, respectively (*p* ≤ 0.0001). On the other hand, LMNB1 expression decreased 0.79-fold following UVB treatment compared to the control group (*p* ≤ 0.0001). In cells pre-treated with mangiferin and then treated with UVB, the expression levels of CDKN2A, CDKN1A, and TP53 were found to be 1.02-, 1.09-, and 1.16-fold, respectively. LMNB1 expression was determined to be 1.10-fold, and no statistically significant difference was found compared to untreated HDF cells (ns) (Figure 4).

### 3.5. Protective Effects of Mangiferin Against UVB-Induced Extracellular Matrix Damage in HDF Cells

**Figure 5:**
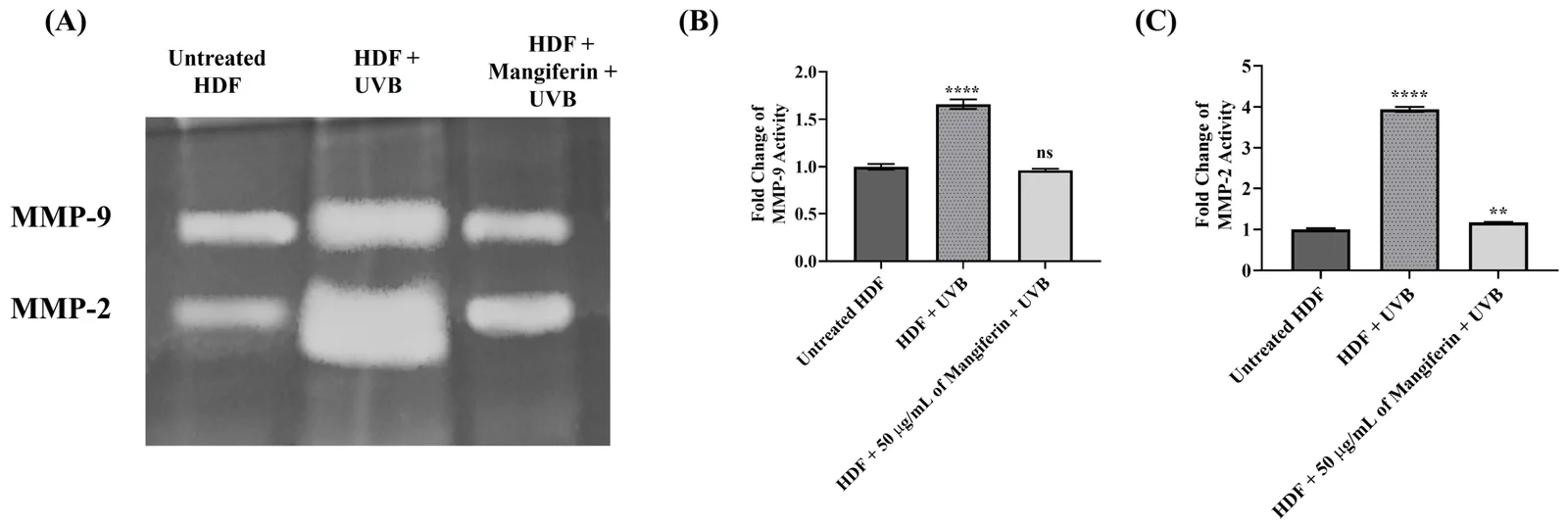
Effects of mangiferin on UVB-induced matrix metalloproteinase (MMP) activity in human dermal fibroblasts (HDFs). (**A**) Representative gelatin zymography gel images showing MMP-9 and MMP-2 activities in untreated HDFs, UVB-exposed HDFs, and mangiferin-pretreated UVB-exposed HDFs. (**B**) Quantitative densitometric analysis of MMP-9 activity. (**C**) Quantitative densitometric analysis of MMP-2 activity. Statistical analysis was performed using one-way ANOVA followed by Tukey’s multiple comparisons test. ns, not significant; ** *p* ≤ 0.01, **** *p* ≤ 0.0001 versus untreated HDF cells.

The protective effects of mangiferin on UVB-induced extracellular matrix (ECM) disruption were investigated by evaluating the activities of MMP-2 and MMP-9 using gelatin zymography (Figure 5). Representative gelatin zymography images are shown in Figure 5A. The lytic white bands observed in the zymograms represent the active MMP-2 and MMP-9 activities. Quantitative results obtained from the densitometric analysis of these bands are presented in Figure 5B for MMP-9 and Figure 5C for MMP-2, respectively. Compared to untreated HDF cells, UVB exposure was found to increase the activities of MMP-2 and MMP-9 by 3.94-fold and 1.66-fold, respectively (*p* ≤ 0.0001). UVB application was observed to cause a particularly significant increase in MMP-2 activity. On the other hand, it was determined that MMP-2 and MMP-9 activities were significantly suppressed in cells exposed to UVB following pre-treatment with mangiferin. Compared with the UVB group, mangiferin treatment significantly reduced MMP-2 and MMP-9 activities by approximately 70.3% and 42.1%, respectively (*p* ≤ 0.0001). These findings suggest that mangiferin may contribute to the preservation of extracellular matrix integrity by suppressing the activation of matrix metalloproteinases involved in UVB-induced ECM degradation.

### 3.6. Inhibitory Activity of Mangiferin on ECM-Degrading Enzymes

**Figure 6:**
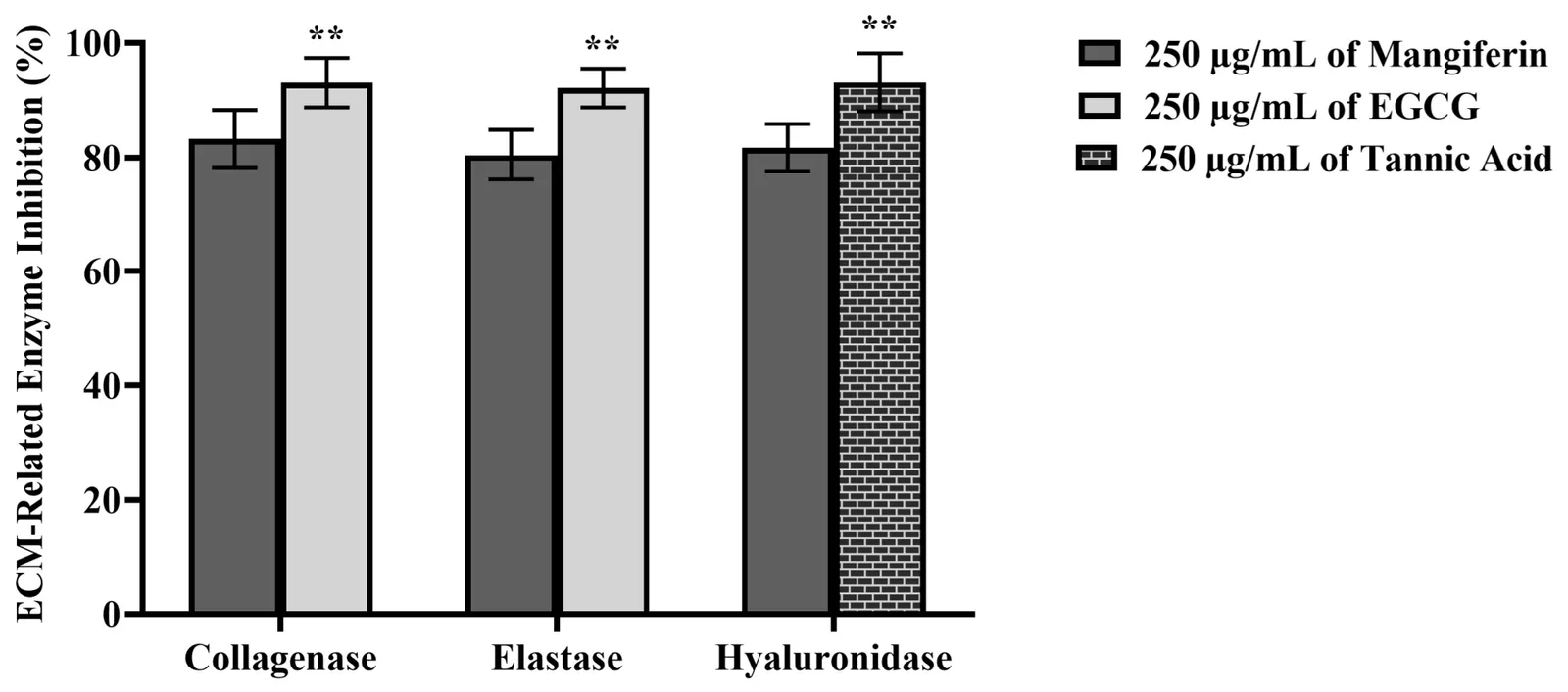
Inhibitory effects of mangiferin on extracellular matrix (ECM)-related enzymes. The inhibitory activities of mangiferin against collagenase, elastase, and hyaluronidase were evaluated and compared with the respective positive controls. EGCG was used as the positive control for collagenase and elastase inhibition, whereas tannic acid was used as the positive control for hyaluronidase inhibition. Enzyme inhibition was expressed as percentage inhibition (%). Statistical analysis was performed using one-way ANOVA followed by Tukey’s multiple comparisons test. ** *p* ≤ 0.01 versus mangiferin-treated samples.

The inhibitory effects of mangiferin on collagenase, elastase, and hyaluronidase enzymes, which play a role in maintaining extracellular matrix (ECM) integrity, were evaluated using spectrophotometric methods (Figure 6). At the predefined cell-free screening concentration of 250 µg/mL, mangiferin inhibited collagenase, elastase, and hyaluronidase activities by 83.27% ± 5.05, 80.46% ± 4.40, and 81.73% ± 4.16, respectively. These findings represent single-concentration measurements of direct biochemical enzyme-inhibitory potential and should not be interpreted as IC_50_ values, complete concentration response profiles, or enzyme-kinetic parameters. The inhibition rates of EGCG, used as a positive control, on collagenase and elastase were determined as 93.11% ± 4.33 and 92.17% ± 3.38, respectively, while tannic acid inhibited hyaluronidase by 93.15% ± 5.06 [22]. The findings revealed that mangiferin exhibited strong inhibitory activity on key enzymes involved in ECM degradation and could contribute to the maintenance of extracellular matrix homeostasis (Figure 6).

## 4. Discussion

In recent years, there has been a growing interest in natural antioxidants in photoaging research. The main reason for this is that excessive reactive oxygen species (ROS) production is at the starting point of UVB-induced skin damage [25,26,27]. Reactive species such as superoxide anion (O_2_•^−^), hydroxyl radical (•OH), and hydrogen peroxide (H_2_O_2_), formed following UVB exposure, not only cause direct oxidative damage to lipids, proteins, and DNA, but also trigger the activation of numerous cellular signaling pathways associated with aging. Therefore, suppressing oxidative stress is considered one of the most important targets that can be addressed in the early stages of the photoaging process [25,26,27].

Mangiferin is a naturally occurring C-glycosylated xanthon derivative found in various plants, primarily Mangifera indica. Thanks to its numerous hydroxyl groups, it exhibits strong electron and hydrogen donor properties, playing an effective role in the neutralization of free radicals [18,19]. However, the effects of mangiferin are not limited solely to direct ROS scavenging. The literature reports that this compound activates Nrf2-mediated antioxidant defense mechanisms, maintains cellular redox balance, and suppresses downstream consequences of oxidative stress [18,19,28]. Therefore, in the present study, mangiferin was evaluated as a potential natural agent that can comprehensively target UVB-induced photoaging mechanisms. In this study, intracellular ROS levels were significantly increased after UVB application, while pre-treatment with mangiferin significantly suppressed ROS formation, as shown in Figure 2, which is consistent with the literature. This finding was critically important in interpreting other molecular findings obtained from the study. This is because UVB-induced oxidative stress is not only the cause of cellular damage but also the initiator of numerous signaling pathways that regulate inflammation, ECM degradation, DNA damage, and cellular aging [18,19,28]. In other words, ROS production is the starting point of numerous pathological events that are central to and reinforce each other in the photoaging process. The RAGE/NF-κB/p38 MAPK axis is the most prominent of these pathways. Although RAGE was initially identified for advanced glycation end products (AGEs), it is now considered a multi-ligand receptor that can be activated by numerous endogenous ligands that arise during oxidative stress [8]. Increased ROS levels due to UVB increase RAGE expression, and activated RAGE stimulates NF-κB and MAPK family members, ensuring the continuity of inflammatory signaling. Thus, a positive feedback loop is created between oxidative stress and inflammation [8,29]. The results presented in Figure 3 showed that UVB exposure was accompanied by increased RAGE, p-NF-κB, and p-p38 MAPK levels, whereas mangiferin pretreatment markedly attenuated each of these changes. This coordinated pattern suggests that modulation of RAGE/NF-κB/p38 MAPK-associated stress signaling may contribute to the protective response observed following mangiferin pretreatment. Activation of NF-κB plays a central role in the inflammatory component of the photoaging process. Activated NF-κB is transported to the nucleus, increasing the expression of genes encoding TNF-α, IL-1β, IL-6, and various matrix metalloproteinases [27]. Therefore, NF-κB is considered one of the key transcription factors that drive not only inflammation but also ECM degradation. Similarly, p38 MAPK is also a key regulator of UVB-induced stress responses. Activated by oxidative stress, p38 MAPK stimulates transcription factors such as AP-1 and NF-κB, increasing MMP synthesis and contributing to the disruption of ECM integrity [27,30]. The reduction in p-p38 MAPK level following mangiferin pretreatment is consistent with attenuation of UVB-associated stress signaling. However, because pathway-specific inhibition or genetic manipulation was not performed, these findings are interpreted as evidence of coordinated pathway-associated modulation rather than direct causal proof that p38 MAPK mediates all downstream effects of mangiferin.

Maintaining ECM homeostasis is one of the key goals of anti-photoaging approaches. This is because clinically observed wrinkle formation, loss of elasticity, and dermal thinning are fundamentally caused by the progressive deterioration of collagen and elastin networks [16,31]. However, it is now accepted that ECM degradation is not merely a structural change, but also a dynamic biological process affecting inflammation, cellular senescence, and tissue remodeling [17]. MMP-2 and MMP-9 are among the gelatinases most frequently associated with the photoaging process. These enzymes are responsible for the degradation of denatured collagen, gelatin, and various matrix proteins, which are important components of the basal membrane and ECM [17]. While MMP activity is essential for tissue regeneration and wound healing under physiological conditions, overexpression and activation following UVB exposure disrupt ECM turnover balance, leading to pathological matrix degradation [16,31,32]. As a result, the collagen network weakens, dermal organization is disrupted, and the clinical signs of photoaging, such as wrinkle formation, loss of elasticity, and dermal thinning, occur [1,14,16]. Furthermore, it has been reported that increased MMP activity in chronic UVB exposure is not limited solely to ECM degradation; it may also contribute to microenvironmental changes associated with cell migration, invasion, and photocarcinogenesis [33]. The findings presented in Figure 5 show a significant increase in MMP-2 and MMP-9 activities after UVB application, while pre-treatment with mangiferin significantly suppressed the activity of both enzymes. Importantly, instead of reducing MMP activities below basal levels, mangiferin brought them to levels close to the control group by limiting UVB-induced overactivation. This finding suggests that mangiferin can suppress UVB-induced proteolytic stress without hindering physiological ECM turnover and contributes to the maintenance of ECM homeostasis. More importantly, the reduction in MMP activities, when considered together with the observed suppression of RAGE, p-NF-κB, and p-p38 MAPK levels, suggests that the ECM-protective effect of mangiferin may stem from the inhibition of upstream inflammatory and stress response signaling. This suggests that ECM protection is not only an enzymatic outcome but also a downstream outcome of the suppression of the ROS-RAGE-NF-κB/MAPK axis. In addition, the results presented in Figure 6 demonstrate that collagenase, elastase, and hyaluronidase activities, which act alongside MMPs in ECM remodeling and photoaging, were significantly inhibited by mangiferin. The cellular and cell-free experiments provide complementary information concerning the potential effects of mangiferin on ECM homeostasis. Gelatin zymography demonstrated that 50 µg/mL mangiferin attenuated UVB-induced MMP-9 and MMP-2 activities in living HDFs, whereas the cell-free assays demonstrated direct inhibitory potential against collagenase, elastase, and hyaluronidase at the predefined screening concentration of 250 µg/mL. The cell-free findings therefore provide preliminary biochemical evidence complementary to the suppression of UVB-induced gelatinase activity observed under cellular conditions. Together, these findings suggest that mangiferin may contribute to ECM homeostasis through complementary cellular and direct biochemical effects. Collagenase, elastase, and hyaluronidase are major ECM-degrading enzymes responsible for the degradation of collagen fibrils, elastic fibers, and hyaluronic acid, respectively. Increased collagenase activity reduces dermal collagen reserves, elevated elastase activity promotes disruption of the elastic fiber network, and increased hyaluronidase activity decreases the water-holding capacity of the dermal matrix [16,17,31,32]. Consequently, these processes play a significant role in the development of photoaging symptoms such as wrinkle formation, decreased skin elasticity, and skin dryness. The fact that mangiferin suppresses collagenase, elastase, and hyaluronidase activities indicates that the ECM-protective effect is not limited to the suppression of MMP-2 and MMP-9. These findings suggest that mangiferin maintains ECM homeostasis through two complementary mechanisms. The first mechanism is the reduction in MMP activation through the suppression of ROS production and the associated RAGE/NF-κB/p38 MAPK signaling [18,19,28].

Another important outcome of UVB-induced oxidative stress is the disruption of DNA integrity. Excessive ROS production following UVB exposure activates the DNA damage response by causing DNA base modifications, single-strand and double-strand breaks [10,11,34]. γ-H2AX, one of the early biomarkers of this damage, is particularly important because it indicates the formation of double-strand breaks. Under physiological conditions, DNA repair mechanisms can reverse most of this damage. However, in cases of chronic UVB exposure, DNA damage can become permanent and genomic instability can develop [13,35,36]. Therefore, UVB-induced DNA damage is critically important not only in terms of photoaging but also in terms of photocarcinogenesis. Genomic instability resulting from chronic oxidative stress, inflammation, and inadequate DNA repair can create favorable conditions for mutation accumulation and cellular transformation in the long term [11,36]. Within the scope of this study, the Western blot results shown in Figure 3 indicate that the increase in γ-H2AX detected in the UVB group demonstrates that significant DNA damage developed in fibroblasts. In contrast, the return of γ-H2AX protein levels toward the untreated control value following mangiferin pretreatment suggests marked attenuation of the UVB-induced DNA damage response. This finding suggests that mangiferin can be considered not only as an antioxidant but also as a protective agent that can limit the downstream consequences of UVB-induced genomic stress. The relationship between DNA damage response and cellular senescence is largely regulated by the p53/p21 and p16INK4a/Rb axes [15,16,37]. UVB-induced DNA double-strand breaks enhance the generation of γ-H2AX, a classic marker of DNA damage, to activate the ATM/ATR-dependent DNA damage response signaling cascade [38]. During this process, activated p53 acts as one of the key tumor suppressor proteins for maintaining genomic integrity and increases the expression of p21, encoded by the CDKN1A gene [38]. p21 inhibits cyclin-dependent kinases, thereby halting the cell cycle, particularly at the G1/S and G2/M checkpoints, and providing time for DNA repair [37,39]. This mechanism constitutes one of the key cellular safety checkpoints that prevents the proliferation of cells carrying damaged DNA. However, if DNA damage becomes permanent, this temporary protective response transforms into an irreversible cellular senescence program [16,35,37,39]. Another senescence marker that plays an important role in the photoaging process is p16INK4a, encoded by CDKN2A. p16 suppresses CDK4/6 activity, keeping the retinoblastoma (Rb) protein active and contributing to the permanent arrest of the cell cycle in the G1 phase [12,16]. Therefore, the p53/p21 axis represents more of an early response to DNA damage, while p16 activation is associated with a more persistent senescence phenotype [37]. In chronic UVB exposure, simultaneous activation of both pathways leads to decreased fibroblast proliferation and the accumulation of senescent cells. LMNB1 is another important molecule associated with senescence; it is one of the essential structural components of the nuclear lamina and plays a critical role in maintaining nuclear integrity [14]. LMNB1 loss contributes to disruption of nuclear architecture, changes in chromatin organization, and the emergence of age-related gene expression programs. Therefore, LMNB1 reduction, along with p16 and p21 increase, is currently considered one of the reliable biomarkers of senescence [14]. The coordinated increase in γ-H2AX protein level and TP53, CDKN1A, and CDKN2A expression, together with reduced LMNB1 expression, is consistent with activation of a DNA damage-associated senescence molecular response in UVB-exposed HDFs. Conversely, attenuation of γ-H2AX protein levels together with normalization of TP53, CDKN1A, CDKN2A, and LMNB1 expression following mangiferin pre-treatment suggests modulation of the UVB-induced senescence-associated molecular response. This finding suggests that mangiferin may be a versatile photoprotective agent contributing to the preservation of genomic stability and nuclear integrity during the photoaging process. Overall, the principal contribution of the present study is the integrated demonstration that mangiferin pretreatment was associated with coordinated attenuation of UVB-induced ROS accumulation, RAGE/p-NF-κB/p-p38 MAPK-related changes, γ-H2AX protein level, senescence-associated transcriptional alterations, and cellular and cell-free ECM-degrading activities in an HDF-based experimental model. These parallel changes do not establish direct causality among all measured pathways, because pathway-specific inhibition or genetic manipulation was not performed. Nevertheless, the findings extend the existing mangiferin literature by linking previously separate oxidative, stress-signaling, DNA damage, senescence-associated, and ECM-related endpoints within a single experimental framework. This integrated molecular profile suggests that mangiferin may act as a multi-level photoprotective compound against UVB-induced alterations in dermal fibroblasts.

## 5. Conclusions

This study demonstrated that mangiferin exhibits a multifaceted protective effect in a UVB-induced human dermal fibroblast photoaging model. The findings showed that UVB exposure increases intracellular ROS production, activating the RAGE/NF-κB/p38 MAPK signaling axis, resulting in DNA damage, cellular senescence, and extracellular matrix degradation. In contrast, mangiferin application significantly reduced intracellular oxidative stress, suppressed RAGE/NF-κB/p38 MAPK activation, and limited the γ-H2AX-mediated DNA damage response. Furthermore, mangiferin decreased the expression of senescence-related CDKN2A, CDKN1A, and TP53 genes while preserving LMNB1 levels, thus contributing to the prevention of UVB-induced cellular senescence. When evaluated in terms of extracellular matrix homeostasis, mangiferin both suppressed MMP-2 and MMP-9 activities and showed a strong inhibitory effect on collagenase, elastase, and hyaluronidase enzymes. These results reveal that mangiferin is not only an antioxidant that regulates intracellular signaling pathways but also a multi-targeted photoprotective agent that maintains ECM integrity. Overall, this study shows that mangiferin suppresses the UVB-induced photoaging process through interconnected molecular mechanisms encompassing oxidative stress, inflammation, DNA damage, cellular senescence, and matrix degradation. This multifaceted biological activity profile makes mangiferin a promising natural active ingredient for the future development of anti-photoaging dermocosmetic and dermatological products. In particular, its evaluation in topical creams, serums, nanoemulsions, and other advanced dermal carrier systems could contribute to the development of new strategies for preventing UVB-induced skin aging. In accordance with the molecular findings of the present study, future investigations integrating SA-β-gal staining, cell-cycle and proliferation analyses, LMNB1 immunofluorescence, senescence-associated protein measurements, and SASP profiling may provide a more comprehensive molecular and phenotypic characterization of the effects of mangiferin on UVB-induced cellular senescence in dermal fibroblasts. In addition, validation of these findings using three-dimensional human skin models, ex vivo human skin systems, and clinical investigations remains necessary to establish the translational potential of mangiferin.

## Data Availability

The original contributions presented in this study are included in the article. Further inquiries can be directed to the corresponding author.

## Abbreviations

The following abbreviations are used in this manuscript:

AGE	Advanced Glycation End Product
AP-1	Activator Protein-1
ECM	Extracellular Matrix
HDF	Human Dermal Fibroblast
MMP	Matrix Metalloproteinase
NF-κB	Nuclear Factor Kappa B
RAGE	Receptor For Advanced Glycation End Products
ROS	Reactive Oxygen Species
γ-H2AX	Phosphorylated Histone H2AX
TNF-α	Tumor Necrosis Factor Alpha
TP53	Tumor Protein p53
Nrf2	Nuclear Factor Erythroid 2-Related Factor 2
CDKN1A	Cyclin-Dependent Kinase Inhibitor 1A
CDKN2A	Cyclin-Dependent Kinase Inhibitor 2A

## References

[1] Hussein R.S., Bin Dayel S., Abahussein O., El-Sherbiny A.A. (2025). Influences on skin and intrinsic aging: Biological, environmental, and therapeutic insights. J. Cosmet. Dermatol..

[2] Zhang J., Jia X., Fu Q., Bai X., Wang J., Qin Y., Yang J., Qi F., Pan Y. (2026). Ultraviolet radiation reshapes the transcriptomic landscape of human skin aging: Insights from a multi-age comparative study. J. Photochem. Photobiol. B Biol..

[3] Huang Y., Song J., Wang Z., Zhu S., Hu Y., Gan X., Luo S., He Q., Zhang L., Wang Q. (2025). Antiphotoaging effects of a group of antioxidant peptides through downregulating matrix metalloproteinases and inflammation factors. Front. Cell Dev. Biol..

[4] Fisher G.J., Wang B., Cui Y., Shi M., Zhao Y., Quan T., Voorhees J.J. (2023). Skin aging from the perspective of dermal fibroblasts: The interplay between the adaptation to the extracellular matrix microenvironment and cell autonomous processes. J. Cell Commun. Signal..

[5] Lin W.-T., Chen Y.-J., Kuo H.-N., Kumar S., Abomughaid M.M., Senthil Kumar K.J. (2025). Ultraviolet B-induced oxidative damage in human skin keratinocytes is alleviated by *Pinus morrisonicola* leaf essential oil through activation of the Nrf2-dependent antioxidant defense system. Redox Rep..

[6] Schieber M., Chandel N.S. (2014). ROS function in redox signaling and oxidative stress. Curr. Biol..

[7] Naharro-Rodriguez J., Bacci S., Hernandez-Bule M.L., Perez-Gonzalez A., Fernandez-Guarino M. (2025). Decoding skin aging: A review of mechanisms, markers, and modern therapies. Cosmetics.

[8] Radziszewski M., Galus R., Łuszczyński K., Winiarski S., Wąsowski D., Malejczyk J., Włodarski P., Ścieżyńska A. (2024). The RAGE pathway in skin pathology development: A comprehensive review of its role and therapeutic potential. Int. J. Mol. Sci..

[9] Umar S.A., Tasduq S.A. (2020). Integrating DNA damage response and autophagy signalling axis in ultraviolet-B induced skin photo-damage: A positive association in protecting cells against genotoxic stress. RSC Adv..

[10] Schütz C.S., Stope M.B., Bekeschus S. (2021). H2AX phosphorylation in oxidative stress and risk assessment in plasma medicine. Oxid. Med. Cell. Longev..

[11] Podhorecka M., Skladanowski A., Bozko P. (2010). H2AX phosphorylation: Its role in DNA damage response and cancer therapy. J. Nucleic Acids.

[12] Kelm R.C., Murphrey M.B. (2026). The impact of lasers and energy-based devices on cellular senescence: A systematic review. Lasers Surg. Med..

[13] Wen S.-Y., Ng S.-C., Chiu Y.-T., Tai P.-Y., Chen T.-J., Chen C.-J., Huang C.-Y., Kuo W.-W. (2024). Enhanced SIRT1 activity by galangin mitigates UVB-induced senescence in dermal fibroblasts via p53 acetylation regulation and activation. J. Agric. Food Chem..

[14] Wang A.S., Ong P.F., Chojnowski A., Clavel C., Dreesen O. (2017). Loss of Lamin B1 is a biomarker to quantify cellular senescence in photoaged skin. Sci. Rep..

[15] Jipu R., Serban I.L., Goriuc A., Jipu A.G., Luchian I., Amititeloaie C., Tarniceriu C.C., Hurjui I., Butnaru O.M., Hurjui L.L. (2025). Targeting dermal fibroblast senescence: From cellular plasticity to anti-aging therapies. Biomedicines.

[16] Wang A.S., Dreesen O. (2018). Biomarkers of cellular senescence and skin aging. Front. Genet..

[17] Shin J.-W., Kwon S.-H., Choi J.-Y., Na J.-I., Huh C.-H., Choi H.-R., Park K.-C. (2019). Molecular mechanisms of dermal aging and antiaging approaches. Int. J. Mol. Sci..

[18] Al-Madhagi H. (2025). From nature to nanotechnology: The bioactivities of mangiferin explored. Nanotechnol. Sci. Appl..

[19] Imran M., Arshad M.S., Butt M.S., Kwon J.-H., Arshad M.U., Sultan M.T. (2017). Mangiferin: A natural miracle bioactive compound against lifestyle related disorders. Lipids Health Dis..

[20] Kurt-Celep İ., Celep E., Akyüz S., İnan Y., Barak T.H., Akaydın G., Telci D., Yesilada E. (2020). *Hypericum olympicum* L. recovers DNA damage and prevents MMP-9 activation induced by UVB in human dermal fibroblasts. J. Ethnopharmacol..

[21] Kurt-Celep İ., Ahmed S., Cetiz M.V., Uba A.I., Ak G., Selvi S., Emir A., Dall’Acqua S., Zengin G. (2025). Revealing a gold mine of bioactive compounds from natural sources using in vitro, in silico, and network pharmacology: A case study on *Cachrys cristata*. Arch. Pharm..

[22] Kurt-Celep İ., Cusumano G., Flores G.A., Peron G., Senkardes İ., Angelini P., Emiliani C., Uba A.I., Zengin G. (2026). Harnessing the anti-inflammatory, skin-protective, and antioxidant potential of *Epilobium dodonaei* extracts using in vitro and in silico approaches. RSC Adv..

[23] Kurt-Celep İ., Kilinc A.N., Griffin M., Telci D. (2022). Nitrosylation of tissue transglutaminase enhances fibroblast migration and regulates MMP activation. Matrix Biol..

[24] Parusnath M., Naidoo Y., Singh M. (2026). Antioxidant activity, cytotoxicity and antibacterial activity of *Solanum mauritianum* Scop. (Solanaceae) extracts. S. Afr. J. Bot..

[25] Gulcin İ. (2025). Antioxidants: A comprehensive review. Arch. Toxicol..

[26] Calvo M.J., Navarro C., Durán P., Galan-Freyle N.J., Parra Hernández L.A., Pacheco-Londoño L.C., Castelanich D., Bermúdez V., Chacin M. (2024). Antioxidants in photoaging: From molecular insights to clinical applications. Int. J. Mol. Sci..

[27] Lei X. (2025). Mechanisms and therapeutic roles of medicinal plants in skin photoaging. Clin. Cosmet. Investig. Dermatol..

[28] Zhang B.-P., Zhao J., Li S.-S., Yang L.-J., Zeng L.-L., Chen Y., Fang J. (2014). Mangiferin activates Nrf2-antioxidant response element signaling without reducing the sensitivity to etoposide of human myeloid leukemia cells in vitro. Acta Pharmacol. Sin..

[29] Zhu P., Ren M., Yang C., Hu Y.-X., Ran J.-M., Yan L. (2012). Involvement of RAGE, MAPK and NF-κB pathways in AGEs-induced MMP-9 activation in HaCaT keratinocytes. Exp. Dermatol..

[30] Kwon K.-R., Alam M.B., Park J.-H., Kim T.-H., Lee S.-H. (2019). Attenuation of UVB-induced photo-aging by polyphenolic-rich *Spatholobus suberectus* stem extract via modulation of MAPK/AP-1/MMPs signaling in human keratinocytes. Nutrients.

[31] Wang X., Zhou F., Bie F., Xiong Y., Li Z., Liu X., Mo X., Zhao J., Zhang Z., Xie J. (2026). A multifunctional nano-hydrogel attenuates skin photoaging via regulating ECM microenvironment homeostasis. Mater. Today Bio.

[32] Wei M., He X., Liu N., Deng H. (2024). Role of reactive oxygen species in ultraviolet-induced photodamage of the skin. Cell Div..

[33] Pittayapruek P., Meephansan J., Prapapan O., Komine M., Ohtsuki M. (2016). Role of matrix metalloproteinases in photoaging and photocarcinogenesis. Int. J. Mol. Sci..

[34] Kciuk M., Marciniak B., Mojzych M., Kontek R. (2020). Focus on UV-induced DNA damage and repair—Disease relevance and protective strategies. Int. J. Mol. Sci..

[35] Toyoda T., Akagi J.-I., Cho Y.-M., Mizuta Y., Onami S., Suzuki I., Ogawa K. (2013). Detection of γ-H2AX, a biomarker for DNA double-strand breaks, in urinary bladders of N-butyl-N-(4-hydroxybutyl)-nitrosamine-treated rats. J. Toxicol. Pathol..

[36] Colombowala A., Aruna K. (2022). Phosphorylated H2AX: Prospective role in DNA damage responses and a credible tool for translational cancer research. J. Appl. Biotechnol. Rep..

[37] Yan J., Chen S., Yi Z., Zhao R., Zhu J., Ding S., Wu J. (2024). The role of p21 in cellular senescence and aging-related diseases. Mol. Cells.

[38] Oh K.-S., Bustin M., Mazur S.J., Appella E., Kraemer K.H. (2011). UV-Induced Histone H2AX phosphorylation and DNA damage related proteins accumulate and persist in nucleotide excision repair-deficient XP-B cells. DNA Repair.

[39] Al Bitar S., Gali-Muhtasib H. (2019). The role of the cyclin dependent kinase inhibitor p21cip1/waf1 in targeting cancer: Molecular mechanisms and novel therapeutics. Cancers.

